# 3 Tesla breast MR imaging as a problem-solving tool: Diagnostic performance and incidental lesions

**DOI:** 10.1371/journal.pone.0190287

**Published:** 2018-01-02

**Authors:** Claudio Spick, Dieter H. M. Szolar, Klaus W. Preidler, Pia Reittner, Katharina Rauch, Peter Brader, Manfred Tillich, Pascal A. Baltzer

**Affiliations:** 1 Department of Biomedical Imaging and Image-guided Therapy, Medical University of Vienna (AKH), Vienna, Austria; 2 Diagnostikum Graz, Graz, Austria; University of Graz, AUSTRIA

## Abstract

**Purpose:**

To investigate the diagnostic performance and incidental lesion yield of 3T breast MRI if used as a problem-solving tool.

**Methods:**

This retrospective, IRB-approved, cross-sectional, single-center study comprised 302 consecutive women (mean: 50±12 years; range: 20–79 years) who were undergoing 3T breast MRI between 03/2013-12/2014 for further workup of conventional and clinical breast findings. Images were read by experienced, board-certified radiologists. The reference standard was histopathology or follow-up ≥ two years. Sensitivity, specificity, PPV, and NPV were calculated. Results were stratified by conventional and clinical breast findings.

**Results:**

The reference standard revealed 53 true-positive, 243 true-negative, 20 false-positive, and two false-negative breast MRI findings, resulting in a sensitivity, specificity, PPV, and NPV of 96.4% (53/55), 92.4% (243/263), 72.6% (53/73), and 99.2% (243/245), respectively. In 5.3% (16/302) of all patients, incidental MRI lesions classified BI-RADS 3–5 were detected, 37.5% (6/16) of which were malignant. Breast composition and the imaging findings that had led to referral had no significant influence on the diagnostic performance of breast MR imaging (p>0.05).

**Conclusion:**

3T breast MRI yields excellent diagnostic results if used as a problem-solving tool independent of referral reasons. The number of suspicious incidental lesions detected by MRI is low, but is associated with a substantial malignancy rate.

## Introduction

Mammography and ultrasound are established tests in the diagnosis of breast cancer [1–3]. Still, these imaging modalities regularly yield inconclusive findings where the presence of breast cancer cannot be confirmed or excluded. In these cases, image-guided biopsies are performed to establish a diagnosis. Biopsy, however, is challenging if findings are difficult to localize, as is the case in architectural distortions or diffuse and multiple lesions. In this uncommon but diagnostic challenging setting, breast MR imaging can be used as a problem-solving tool, which can either help guide or avoid biopsies due to its high sensitivity and soft tissue contrast. This indication for MR imaging is, however, controversial in the imaging community [4–7]. While breast MR imaging provides a very high sensitivity and negative predictive value, particularly in non-calcified breast lesions [5,8], a recent meta-analysis highlighted important research gaps: problem-solving definitions are not well-defined and the empirical evidence about specific indications, such as architectural distortions, is sparse [5,9,10]. In addition, it remains unclear whether MR imaging actually facilitates the work-up of inconclusive cases or not. Additional MR imaging findings that are unrelated to the reason for the original referral require further work-up, which is justified if additional cancer lesions are detected, but is ultimately unnecessary in case of benign lesions.

Consequently, the purpose of this study was to evaluate the diagnostic performance and incidental lesion yield of breast MR imaging if used as a problem-solving tool and align these results with original referral reasons.

## Materials and methods

### Case selection and reference standard

Eligible for this retrospective, single-center, IRB-approved study were women who were consecutively referred to our institution (Diagnostikum Graz), an independent cross-sectional imaging centre receiving outpatients from multiple referring physicians. Patients were referred for breast MR examinations due to findings on digital mammography and/or ultrasound between March 2013 and December 2014. Specifically, we included those patients in that according with our national health care system regulations received an interim BI-RADS 0 category (further imaging assessment required) assignment. This problem-solving indication includes a variety of findings such as discrepancies between MG and US such as asymmetric densities without US correlate, lesions with discrepant size in both modalities, lesions with equivocal morphology in either both or one of these modalities and multiple lesions. The indication for MR imaging in this setting is assessed by the representative physicians of the medical authorities, in compliance with national health regulations. This study was conducted according to STARD (**S1 File).**

Included in this study were those women who had a reference standard of either histopathology or imaging follow-up at 24 months. Histopathological diagnosis was established either by image-guided biopsy (ultrasound-guided core biopsy or vacuum-assisted biopsy under mammography/MR imaging guidance) or open surgery. Experienced breast pathologists performed the breast tissue specimen work-up. Lesions classified as benign by imaging or histopathology were followed up by imaging (mammography, ultrasound, or MR imaging, as appropriate) for at least 24 months. Excluded were patients with contraindications against MR imaging or incomplete MR imaging scans.

### MR imaging

MR imaging was performed on a 3T magnet (Magnetom Skyra, Siemens Medical Solutions®, Erlangen, Germany) using the vendor-supplied, 16-channel dedicated breast coil. Breast MR imaging examinations were generally scheduled in the second week of the menstrual cycle in premenopausal women. Menopausal patients who were receiving hormonal replacement therapy were requested to cease treatment one month before the examination [11].

The imaging protocol encompassed an axial T2w-TSE sequence (Turbo Spin Echo DIXON fast, TR 6500 ms, TE 81 ms, flip angle 120°, spatial resolution 4 mm^3^, 35 slices, time of acquisition 2:10 minutes), and a readout-segmented, multi-shot echo planar imaging-based, diffusion-weighted imaging sequence (RESOLVE, TR 5500 ms, TE1 56 ms, TE2 88 ms, b-values 0 and 800 s/mm^2^, spatial resolution 1.9 x 1.9 x 5 mm 18 mm^3^, 28 slices, no interslice gap, three orthogonal directions, one average, acquisition time 3:36 min). The scanner software (Syngo MR E11, release number: N_4VE11C) automatically calculated the Apparent Diffusion Coefficient (ADC) maps. T1-weighted images were acquired as follows: FLASH 3D; SPAIR fat saturation; TR 4.89 ms; TE 1.81 ms; flip angle 10°; spatial resolution 0.9 x 0.9 x 1.8 mm 1.5 mm^3^; and time of acquisition 1:10 minutes per measurement. These were obtained once before and four times after the intravenous injection of 0.1 mmol/kg gadoteridol (Prohance®, BRACCO, Milano, Italy). Between postcontrast measurements 3 and 4, an interleaved, isotropic, high-resolution T1w sequence was acquired (FLASH 3D, SPAIR fat saturation, TR 7.33 ms, TE 3.73 ms, flip angle 15°, spatial resolution 0.9 mm isotropic, time of acquisition 2:26). Image subtractions were calculated in-line by the scanner software (Syngo MR E11, release number: N_4VE11C). Overall acquisition magnet time for this protocol was less than 15 minutes.

### MR image interpretation

All imaging data was read during routine clinical practice by one of four board-certified radiologists with >10 years of experience. Results were saved in a prospectively populated database within our institution´s electronic information system. Image interpretation was performed according to the American College of Radiology BI-RADS® lexicon using morphologic and dynamic enhancement criteria [12] before any histopathological sampling. Signal intensity time curves were measured by Regions-of-Interest placed in the most enhancing part of the lesion [13]. Image interpretation during routine clinical practice considered the results of prior images as this facilitates image interpretation [14]. In addition, lesions showing high ADC values were considered benign as suggested in the literature [15–18]. Lesions without contrast-enhancement on MRI were generally considered benign.

### Data analysis

Data were extracted from our institutional prospectively populated database into a computerized spreadsheet (Excel: Microsoft, Redmond, WA).

Receiver-Operating-Characteristics (ROC) analysis was performed using BI-RADS as the classification variable and final diagnosis, based on the reference standard (benign versus malignant), as the reference variable. Lesions were considered malignant if image-guided biopsy or surgery, or both, confirmed invasive carcinoma or DCIS. The reference standard for benign lesions was histopathology (biopsy and/or surgery) and imaging follow-up of at least two years or only imaging follow-up of at least two years. Histopathological diagnoses were established by board-certified breast pathologists.

MR imaging reading results were compared to reference standard results to calculate true positive (TP), true negative (TN), false positive (FP), and false negative (FN) findings. For this purpose, BI-RADS category assignments 1–3 were considered negative and 4–5 positive. Additional evaluation of diagnostic parameters was performed considering BI-RADS category assignments 1 and 2 negative and 3–5 positive.

The diagnostic parameters sensitivity TP/(TP+FN), specificity TN/(TN+FP), positive predictive value (PPV) TP/(TP+FP), and negative predictive value (NPV) TN/(TN+FN) were stratified by clinical presentation, conventional imaging findings, and breast density, and were compared using McNemar tests. A P-value ≤0.05 was considered to indicate a significant result.

## Results

### Patients and lesions

Of 322 patients, 302 women (mean age, 50±12 years; range, 20–79 years) fulfilled the inclusion criteria, and comprised the study cohort. Twenty patients (6%) with MR imaging BI-RADS 2 (n = 18) and MR imaging BI-RADS 3 (n = 2) ratings were lost to follow-up. Thus, 302 patients were included in the analysis. Indications for the examination are listed in Table 1.

**Table 1:** Summary of the results of this study: MRI findings are stratified by conventional imaging findings, clinical presentation, and ACR breast composition. Resulting cancer prevalence, sensitivity, specificity, positive predictive value (PPV), and negative predictive value (NPV) considering BI-RADS 4 and 5 as positive and BI-RADS 1–3 as negative MRI results.

	Total	TP	TN	FP	FN	Cancer prevalence (%)	Sensitivity (%)	Specificity (%)	PPV (%)	NPV (%)
**All**	318	53	243	20	2	17.3%	96.4% (CI 87.5%-99.6%)	92.4% (CI 88.5%-95.3%)	72.6% (CI 60.9%-82.4%)	99.2% (CI 97.1%-99.8%)
**Index lesion**	302	48	236	17	1	16.2%	98.0% (CI 89.2%-100%)	93.3% (CI 89.5%-96.1%)	73.9% (CI 61.5%-84.0%)	99.6% (CI 97.7%-100%)
**Mammography**										
Mass	163	24	128	10	1	15.3%	96% (CI 79.7%-99.9%)	92.8% (CI 87.1%-96.5%)	70.6% (CI 52.5%-84.9%	99.2% (CI 85.8%-100%
Mass with architectural distortion	19	8	10	1	0	42.1%	100% (CI 63.1%-100%)	90.9% (CI 58.7%-99.8%)	88.9% (CI 51.8%-99.7%)	100% (CI 69.2%-99.8%)
Mass with microcalcifications	18	4	14	0	0	22.2%	100% (CI 39.8%-100%)	100% (CI 78.8%-100%)	100% (CI 39.8%-100%)	100% (CI 76.8%-100%)
Architectural distortion	69	8	55	6	0	11.6%	100% (CI 63.1%-100%)	90.2% (CI 79.8%-96.3%)	97.1% (CI 28.9%-82.3%)	100% (CI 93.5%-100%)
Architectural distortion with micro	7	1	6	0	0	14.3%	100% (CI 2.5%-100%)	100% (CI 54.1%-100%)	100% (CI 2.5%-100%)	100% (CI 54.1%-100%)
Microcalcifications	26	3	23	0	0	11.5%	100% (CI 29.2%-100%)	100% (CI 85.2%-100%)	100% (CI 29.2%-100%)	100% (CI 85.2%-100%)
**Clinical presentation**										
Palpable	84	22	54	8	0	26.2%	100% (CI 84.6%-100%)	87.1% (CI 76.2%-94.26%)	73.3% (CI 59.2%-84%)	100%
Not palpable	218	26	182	9	1	12.4%	96.3% (CI 81.1%-99.9%)	95.3% (CI 91.2%-97.8%)	74.3% (CI 60.2%-84.6%)	99.5% (CI 96.4%-100%)
**Breast composition**										
ACR a	17	3	14	0	0	17.3%	100% (CI 29.2%-100%)	100% (CI 76.8%-100%)	100%	100%
ACR b	89	20	62	6	1	23.6%	95.24% (CI 76.2%-99.9%)	91.2% (CI 81.8%-96.7%)	76.9% (CI 60.7%-87.8%)	98.4% (CI 90.1%-99.8%)
ACR c	153	17	126	10	0	11.1%	100% (CI 80.5%-100%)	92.7% (CI 86.9%-96.4%)	62.9% (CI 48.4%-76%)	100%
ACR d	43	8	34	1	0	18.6%	100% (CI 63.1%-100%)	97.1% (CI 85.1%-99.3%)	88.9% (CI 53.7%-98.2%)	100%
**MRI-only lesion** **BI-RADS 3–5**	16	5	7	3	1	37.5	83.3% (CI 35.9%-99.6%)	70% (CI 34.8%-93.3%)	62.5% (CI 24.5%-91.5%)	87.5% (CI 47.4%-99.7%)

Note: Index lesions refers to the findings that were the reasons for referral to MRI; MRI-only lesions were those lesions additionally detected by MRI and not detected by the initial assessment before MRI; BI-RADS ratings were dichotomized into positive (4/5) and negative (1/2/3) to count true-positive (TP), true-negative (TN), false-positive (FP) and false-negative (FN) lesions, PPV positive predictive value, and NPV negative predictive value. ACR, American College of Radiology.

Breast MR imaging revealed 144 (45.3%) mass lesions that demonstrated a mean size of 15.3 mm ± 14.3 mm (SD; range, 4–95 mm). Further, there were 44 (13.8%) non-mass enhancements with a mean size of 29.2 mm ± 23.1 mm (SD; range, 4–75 mm).

Final lesion diagnoses were malignant in 55 of 318 lesions (44 mass and 11 non-mass), and benign in 263 (100 mass, 33 non-mass lesions, and 130 without contrast-enhancing correlates in MR imaging). Malignant histopathological diagnoses were: invasive ductal carcinoma (IDC) in 43 (35 mass and eight non-mass); and ductal carcinoma in situ (DCIS) in 12 (nine mass and three non-mass) cases. BI-RADS ratings were assigned as follows: BI-RADS 1/2: 184 (0 malignant); BI-RADS 3: 61 (two malignant); BI-RADS 4: 39 (20 malignant); and BI-RADS 5: 34 (33 malignant). Twenty lesions classified as MR imaging BI-RADS 4/5 showed benign histopathology after image-guided biopsy.

The prevalence of malignancy was 17.3% (55 of 318). The malignancy rate tended to be higher in mass lesions (30.6%, 44 of 144) compared to non-mass lesions (25%, 11 of 44) though this was not statistically significant (P = 0.599). None of the 130 conventional findings without an enhancing MR imaging correlate was malignant.

Malignancy rates differed according to clinical and conventional findings (Table 1). Lesions that presented as a mammographic mass with architectural distortion had the highest probability for malignancy (42.1%), while pure architectural distortions and microcalcifications had the lowest malignancy rates (11.6% and 11.5%, respectively). Details on MR imaging results stratified by clinical presentation and imaging findings are given in Table 1.

### Diagnostic performance

**Fig 1:**
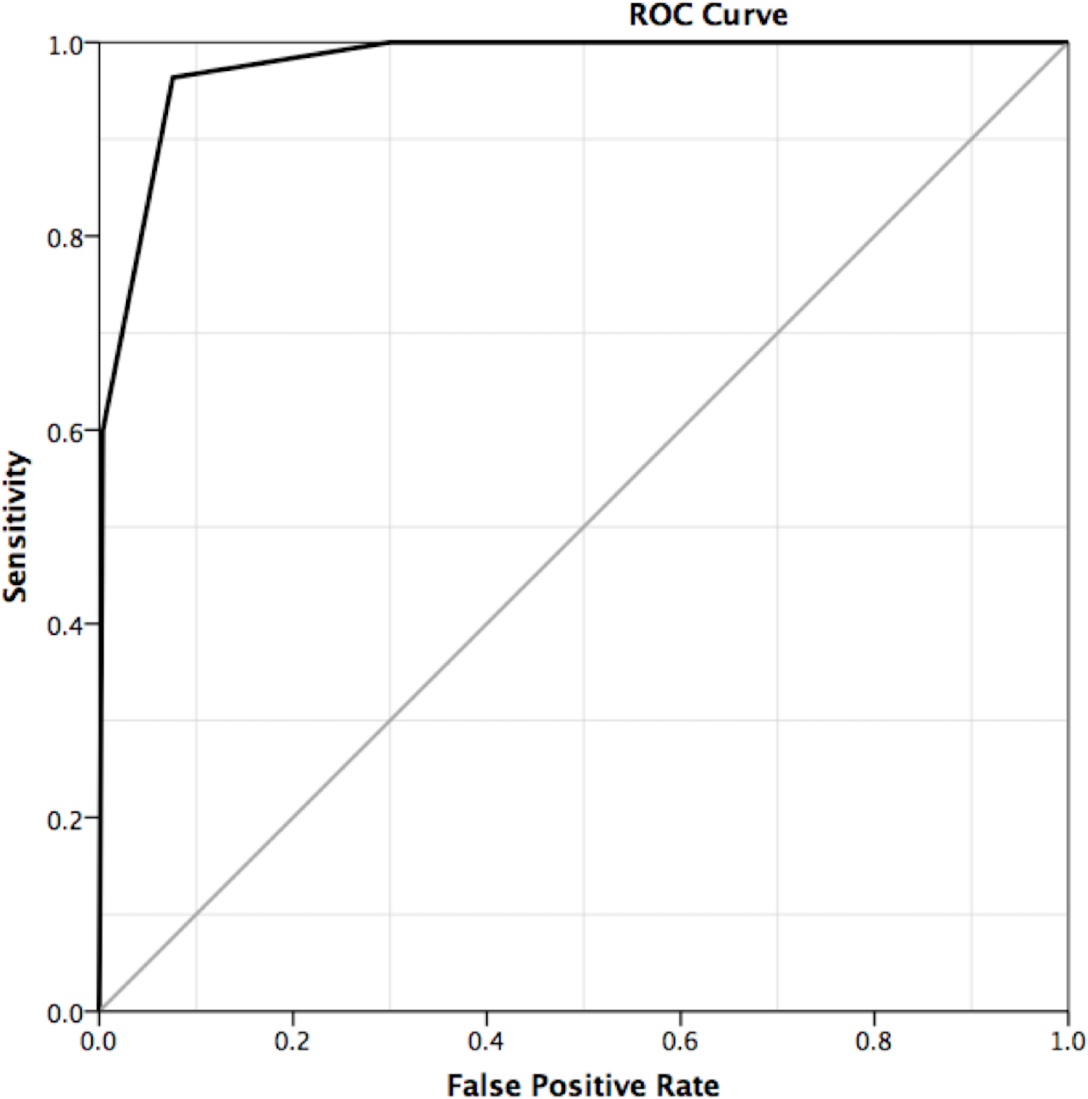
ROC plot of BI-RADS ratings against the reference standard. At a cut-off of >BI-RADS 3, the sensitivity and specificity were 96.4% and 92.4%, respectively. In addition, at a cut-off of >BI-RADS 2 the sensitivity and specificity were 100% and 70.3%, respectively.

**Fig 2:**
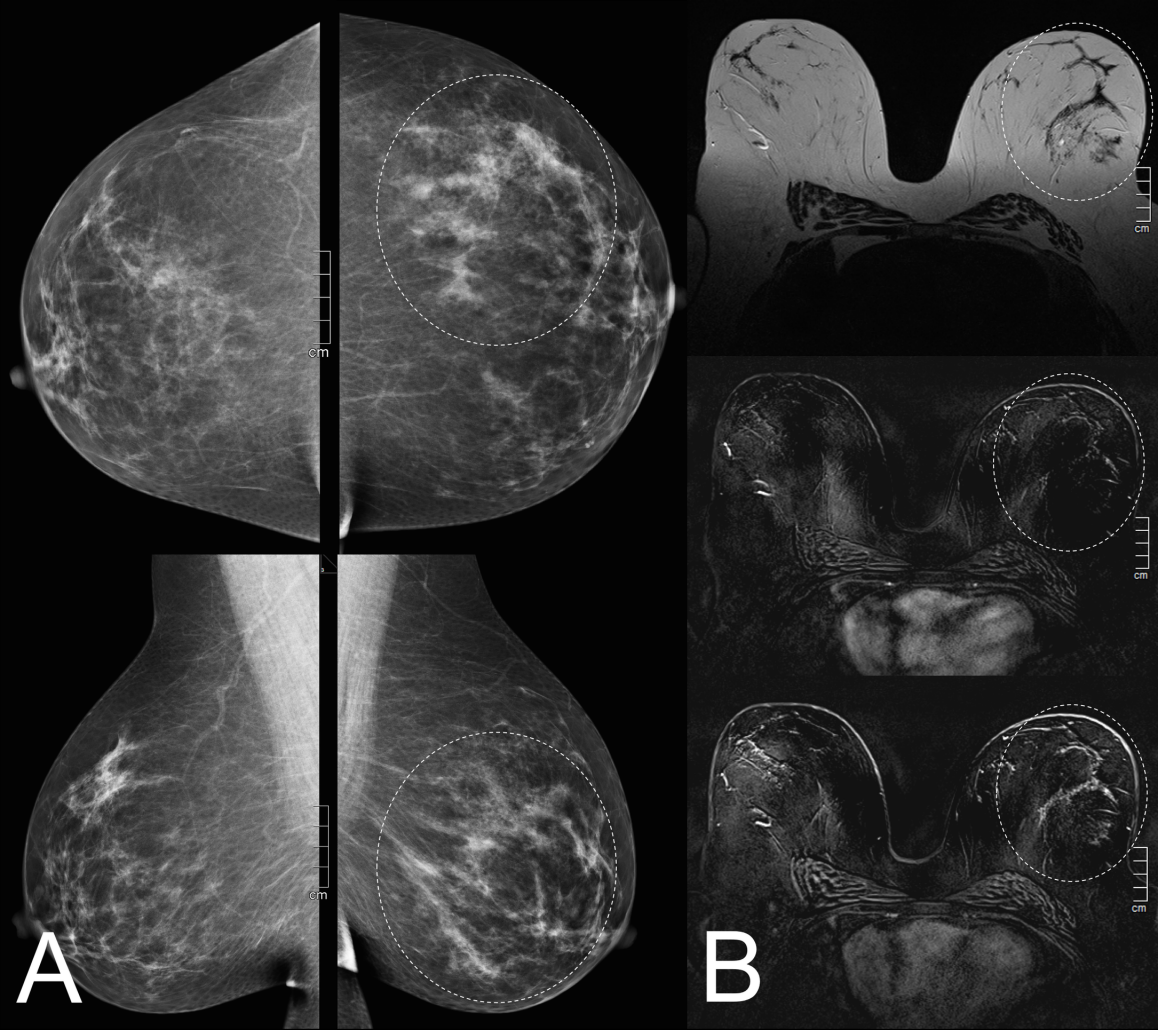
A 52-year-old patient referred for problem-solving due to newly diagnosed architectural distortion in the left breast (A; white circles on mammography images). 3T contrast-enhanced MR imaging (B; top: T2w image, middle: early contrast-enhanced image, bottom: late contrast-enhanced image) shows the architectural distortion (white circle) demonstrating only mild background enhancement. The lesion was classified as BI-RADS 2, definitely benign. Follow-up of two years did not reveal malignancy.

ROC analysis (Fig 1) revealed an area under the ROC curve of 0.977 (95% CI: 0.963–0.992). Considering BI-RADS 4 and 5 malignant and 1–3 benign the reference standard revealed 53 true-positive, 243 true-negative, 20 false-positive, and two false-negative breast MR imaging findings (Fig 2). All underlying data are given in the S2 File. The sensitivity, specificity, and positive and negative predictive values were calculated as: 96.4% (95% CI: 87.5–99.6%), 92.4% (95% CI: 88.5–95.3%), 72.6% (95% CI 60.9–82.4%), and 99.2% (95% CI: 97.1–99.0%), respectively. Upon subgroup analysis, no significant differences in diagnostic parameters were found when stratified by conventional imaging findings, clinical presentation, and breast density (P>0.05). Both false-negative findings were assigned BI-RADS 3 and diagnosed due to changes after short-term follow-up.

Considering BI-RADS 3–5 malignant and 1 and 2 benign the reference standard revealed 55 true-positive, 185 true-negative, 78 false-positive, and no false-negative breast MR imaging findings. The sensitivity, specificity, and positive and negative predictive values were calculated as: 100% (95% CI: 93.5–100%), 70.3% (95% CI: 64.4–75.8%), 41.4% (95% CI 36.9–45.9%), and 100%, respectively.

### Incidental MR imaging lesions

Incidental lesions identified by MR imaging and assigned BI-RADS 3–5 categories were identified in 16 of 302 patients (5.3%). Of these, six lesions (37.5%, 6/16) proved to be malignant. One lesion was assigned a false-negative BI-RADS 3 and the lesion was upgraded upon six-month follow-up, which was initiated due to the MR imaging findings. Consequently, 10.9% (6/55) of all malignant lesions were detected only by MR imaging and presented multifocal or multicentric disease that did not show any conventional imaging correlates. All these lesions measured ≤10 mm. All other lesions detected outside the area of the conventional imaging findings by MR imaging were given BI-RADS category 2 assignments and not considered as incidental lesions for this analysis.

## Discussion

Breast MR imaging yields excellent diagnostic results if used as a problem-solving tool independent of referral reasons. The number of suspicious incidental lesions detected by MR imaging is low, but has a substantial malignancy rate. Our study population included the largest number to date, to our knowledge, of patients undergoing 3 Tesla breast MR imaging for problem-solving. Breast MR imaging had a high negative predictive value of 99.2% and a high PPV of 72.6%. In other words, only three of 10 biopsies recommended based on positive breast MR imaging (BI-RADS 4 and 5) findings were false-positive, and thus, unnecessary, while in eight of 10 patients with a negative MRI result (BI-RADS 1–3), biopsies or further follow-up examinations could be avoided. This came at the cost of two false-negative findings—both of which, however, were detected by short-term follow-up examinations that were initiated due to breast MR imaging BI-RADS 3 lesions, and one of which was detected exclusively by MR imaging. Therefore, all cancers were visualized as enhancing lesions by breast MR imaging. These findings validate that MR imaging is a safe diagnostic instrument if applied as a problem-solving tool in inconclusive cases, as malignancy can be reliably excluded. Furthermore, the number of incidental MR imaging findings that required follow-up or invasive diagnostic procedures was as low as 5.3%, but yielded a substantial malignancy rate of 37.5%. Our multiparametric breast MR imaging protocol is a fast diagnostic test, allowing the examination of up to four patients per hour. Image interpretation and reporting takes only a few minutes and results in high sensitivity and negative predictive values. Thus, breast MR imaging as a problem-solving tool improves patient care by avoiding the anxiety related to follow-up examinations and possibly missed cancer diagnoses.

The indication “problem-solving” for breast MR imaging has been a controversial topic. While a recent meta-analysis corroborated the ability of breast MR imaging to exclude cancer in non-calcified lesions [5], MR imaging might not be as accurate in lesions that present as microcalcifications [8]. Although, based on a rather small study population, our results demonstrated excellent sensitivity and NPV in calcified lesions, a fact that might be explained by the more modern equipment, namely, 3 Tesla in combination with a multichannel coil and high-resolution 3D gradient-echo, T2w, and DWI imaging. None of previous studies investigated this clinical setting exclusively with 3 Tesla breast MR imaging [5,10]. These results are particularly interesting as stereotactically-guided biopsies are more invasive and expensive than US-guided biopsies. Here, MRI would be a valuable test for risk stratification to avoid or guide biopsy in case of multiple or equivocal microcalcifications.

The aforementioned meta-analysis concluded that problem-solving definitions are not well-formulated and the empirical evidence about the performance of breast MR imaging in specific imaging findings that lead to MR imaging, e.g., such as architectural distortions, is sparse [4,5,10,19]. Here, our study directly adds data, as it provides a detailed analysis of the findings that led to the breast MR imaging examination and associates these findings with diagnostic outcomes. Of note, breast MR imaging performance was similar across different indications. Variations were seen regarding malignancy rates and subsequent PPVs, but malignancy could be excluded with high certainty independent of specific indications. Therefore, this study confirms that problem-solving MR imaging is a reliable tool that performs well under varying conditions. Based on our results, we consider all the indications for breast MR imaging investigated in this work as appropriate. Still, we need to stress that breast MR imaging should not generally be used for further evaluation of conventional lesions that can definitely be clarified by US-guided biopsy [9,10,13]. Problem-solving in such lesions would be necessary in case of multiple or difficult to localize lesions that are likely to be missed by immediate biopsy. Although a general application of MR imaging in such lesions has demonstrated excellent diagnostic performance, percutaneous biopsy is readily available, easily tolerated, and leads to a faster definite diagnosis [19, 20, 21].

Breast MR imaging is known to detect more cancers than conventional tests, such as mammography and ultrasound. A common concern among breast imagers is that the inherent higher sensitivity of breast MR imaging will lead to the detection of multiple additional lesions that require further workup. Our data show that this number is, first, as low as 5.3% and, second, entails a substantial malignancy rate of 38%. A recent study showed a similar malignancy rate of 33% (7/22) in suspicious MRI-only lesions [10]. Consequently, our results do not show a relevant number of additional recalls due to the application of MR imaging in the investigated setting. However, suspicious MRI-only lesions warrant further evaluation. Prior studies showed higher rates of incidental recalls between 8.3%-15.6%, with a malignancy rate of these lesions ranging from 0–17% [19,22,23]. The lower recall rate and higher prevalence of malignancy in our cohort is likely due to the exclusive use of 3T multiparametric MR imaging.

We are obliged to mention the limitations of the current study. Twenty patients (6.2%) were lost to follow-up. While this rate is well within the acceptable range, false-negative findings could have been missed, resulting in the low possibility of overestimating sensitivity. The retrospective character of this study did not allow an assessment of inter-reader agreement. However, all examinations were read under routine clinical conditions by experienced breast radiologists. The performance of experienced radiologists has been shown to be superior to that of non-experienced radiologists in the interpretation of breast MR imaging [24]. Further, breast MR images were interpreted considering conventional imaging and clinical findings, an approach that has been recommended, as it improves diagnostic accuracy in non-mass lesions [14]. Accordingly, our audit reflects the actual clinical setting, and thus, provides more representative results as a retrospective reader study under research conditions. Our results were obtained using a fast (<15 min) multiparametric breast MR imaging protocol (T2w, DWI and DCE-MR imaging) at 3 Tesla, using multichannel coil technology. Again, the retrospective character of our study does not allow conclusions on the respective contribution of individual parameters to the final diagnosis. Our experience is, however, in line with prior publications that showed high specificity when T2w or DWI sequences were applied [15,16,18,25].

In conclusion, breast MR imaging yields excellent diagnostic results if used as a problem-solving tool independent of referral reasons. The number of suspicious incidental lesions detected by MR imaging is low, but has a substantial malignancy rate.

## Supporting information

S1 File STARD checklist. (DOC)

S2 File SPSS table of the raw study data. (SAV)
